# The *Hypolimnas misippus* Genome Supports a Common Origin of the W Chromosome in Lepidoptera

**DOI:** 10.1093/gbe/evae215

**Published:** 2024-10-30

**Authors:** Anna Orteu, Shane A McCarthy, Emily A Hornett, Matthew R Gemmell, Louise A Reynolds, Ian A Warren, Ian J Gordon, Gregory D D Hurst, Richard Durbin, Simon H Martin, Chris D Jiggins

**Affiliations:** Department of Zoology, University of Cambridge, Cambridge, UK; Tree of Life Programme, Wellcome Sanger Institute, Hinxton, UK; Tree of Life Programme, Wellcome Sanger Institute, Hinxton, UK; Department of Genetics, University of Cambridge, Cambridge, UK; Institute of Infection, Veterinary and Ecological Science, University of Liverpool, Liverpool, UK; Vector Biology, Liverpool School of Tropical Medicine, Liverpool, UK; Department of Biology, University of Oxford, Oxford, UK; Institute of Infection, Veterinary and Ecological Science, University of Liverpool, Liverpool, UK; Institute of Infection, Veterinary and Ecological Science, University of Liverpool, Liverpool, UK; Department of Zoology, University of Cambridge, Cambridge, UK; Centre of Excellence in Biodiversity, University of Rwanda, Huye, Rwanda; Institute of Infection, Veterinary and Ecological Science, University of Liverpool, Liverpool, UK; Department of Genetics, University of Cambridge, Cambridge, UK; Institute of Evolutionary Biology, University of Edinburgh, Edinburgh, UK; Department of Zoology, University of Cambridge, Cambridge, UK

**Keywords:** sex chromosome evolution, genome assembly, B chromosome, neo-Z chromosome

## Abstract

Moths and butterflies (Lepidoptera) have a heterogametic sex chromosome system with females carrying ZW chromosomes and males ZZ. The lack of W chromosomes in early-diverging lepidopteran lineages has led to the suggestion of an ancestral Z0 system in this clade and a B chromosome origin of the W. This contrasts with the canonical model of W chromosome evolution in which the W would have originated from the same homologous autosomal pair as the Z chromosome. Despite the distinct models proposed, the rapid evolution of the W chromosome has hindered the elucidation of its origin. Here, we present high-quality, chromosome-level genome assemblies of 2 *Hypolimnas* species (*Hypolimnas misippus* and *Hypolimnas bolina*) and use the *H. misippus* assembly to explore the evolution of W chromosomes in butterflies and moths. We show that in *H. misippus*, the W chromosome has higher similarity to the Z chromosome than any other chromosome, which could suggest a possible origin from the same homologous autosome pair as the Z chromosome. However, using genome assemblies of closely related species (ditrysian lineages) containing assembled W chromosomes, we present contrasting evidence suggesting that the W chromosome might have evolved from a B chromosome instead. Crucially, by using a synteny analysis to infer homology, we show that W chromosomes are likely to share a common evolutionary origin in Lepidoptera. This study highlights the difficulty of studying the evolution of W chromosomes and contributes to better understanding its evolutionary origins.

**Significance**

Butterflies and moths have a sex determination system in which females carry 2 different sex chromosomes, Z and W, while males carry 2 copies of the Z. The evolutionary origin of the W chromosome has been elusive, with many possible scenarios being suggested, such as the independent evolution of W chromosomes in many butterfly and moth species. Here, we present genome assemblies of 2 *Hypolimnas* butterfly species and use one of them to shed light on the evolution of the W chromosome. We show that W chromosomes across butterflies and moths are very similar which suggests a shared common origin.

## Introduction

Sex chromosomes are highly variable in eukaryotes and have evolved independently multiple times (Bachtrog et al. 2014, 2011; Beukeboom and Perrin 2014). In animals, there are multiple types of chromosomal sex determination systems, but 2 are predominant: male heterogamety as seen in mammals where males are XY and females XX and female heterogamety as seen in birds where females are ZW and males ZZ (Beukeboom and Perrin 2014). These sex chromosomes (XY and ZW) are often heteromorphic and can potentially originate from different processes. One possibility is that they initially arise from a pair of autosomes that evolve genetic sex determination, and through a process of reduced recombination, sex-specific mutations accumulate (Wright et al. 2016). This reduction in recombination can also lead to gene depletion and the accumulation of repeat and transposable elements (TEs), which are common characteristics of sex-specific (Y or W) chromosomes (Bachtrog et al. 2011; Wright et al. 2016). However, discerning between cause and consequence can be difficult, as repeats and TEs could be enhancing the reduction in recombination rather than resulting from it. Additionally, other autosomes may fuse to the sex chromosomes and become differentiated. Alternatively, the W/Y chromosomes can originate from the recruitment of a B chromosome, which are dispensable chromosomes that are found variably in populations and species (Yoshida et al. 2011).

**Fig. 1.**
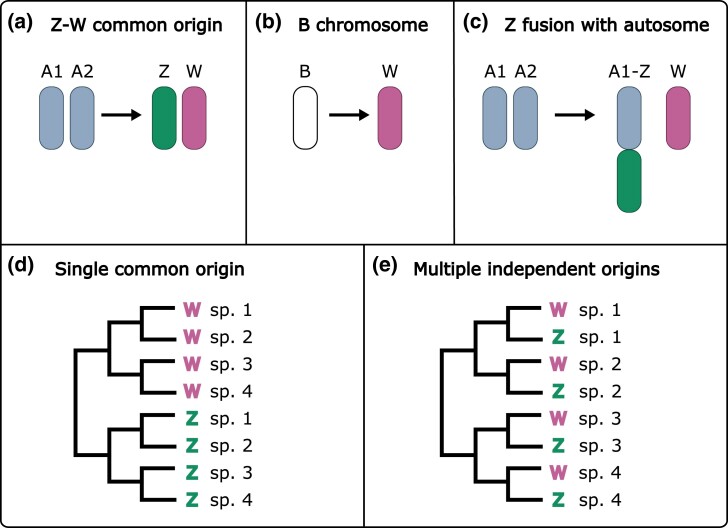
Possible origins of the W chromosome in Lepidoptera. a) One hypothesis on the origin of W chromosomes is that Z and W evolved from the same autosomal pair. b) Another hypothesis is that the W chromosome evolved from B chromosomes, which are nonessential chromosomes that vary in number in populations and species. c) Finally, the inception of W chromosomes could have involved the fusion of the Z chromosome to an autosome and subsequent formation of the W from the autosomal pair. d) In any of these cases, if the W chromosome has originated only once in Lepidoptera, W chromosomes of all species would be more similar to each other than to the Z chromosomes. e) Whereas if they evolved multiple times from the same autosomal pair as the Z, W chromosomes would be more similar to the Z of the same species than to other Ws, while multiple B chromosome recruitments would result in a lack of similarity between Z and W chromosomes as well as among Ws.

Moths and butterflies commonly have a ZW sex chromosome system in which females are heterogametic and show a lack of recombination (Turner and Sheppard 1975). While the Z chromosome has been shown to be highly conserved across the Lepidoptera (Fraïsse et al. 2017), the origin and evolution of the W chromosome remain unclear and several putative origins have been hypothesized. First, some recent evidence has been shown to suggest that the W chromosome originated from the same homologous chromosome pair as the Z (Dai et al. 2022) (Fig. 1a). However, the possible absence of W chromosomes in early-diverging ditrysian lineages (a lepidopteran clade containing all butterflies and most moths) and the deep conservation of the Z chromosome have been suggested to be evidence of an ancestral Z0 sex determination system for Lepidoptera and an origin for the W chromosome that is independent of the Z chromosome (Lukhtanov 2000; Sahara et al. 2012; Dalíková et al. 2017; Fraïsse et al. 2017; Hejníčková et al. 2019). Two main alternative hypotheses have been proposed for the origin of the W chromosome (that is independent of the Z chromosome): evolution from a B chromosome (Lukhtanov 2000; Dalíková et al. 2017; Lewis et al. 2021) (Fig. 1b) or evolution from the homologous pair of an autosome that fused to the Z chromosome (Sahara et al. 2012) (Fig. 1c). Furthermore, and irrespective of the specific origin, the number of evolutionary events leading to the formation of the W chromosome has also been debated. That is whether all W chromosomes in the Lepidoptera share a common evolutionary origin or if their inception was the result of multiple independent events, with the most recent evidence supporting the latter (Lewis et al. 2021; Dai et al. 2022) (Fig. 1d and e).

Despite interest in understanding the evolution of the W chromosome in the Lepidoptera, the absence of high-quality reference genomes containing assembled W chromosomes has limited its study. The elevated repeat and TE content have made its assembly challenging until recently. Long-read sequencing technologies and the decrease in sequencing price have enabled the production of high-quality Lepidopteran genome assemblies containing W chromosomes, which makes it increasingly possible to elucidate the enigmatic origin of the Lepidoptera W chromosome.

*Hypolimnas* butterflies, commonly known as eggflies, are a phenotypically diverse genus that has served as a model for the study of ecology and evolutionary biology. Many *Hypolimnas* species are mimics of toxic species, which has shaped the diversification of wing color pattern in the genus (Vane-Wright et al. 1977). Historically, 2 species, *Hypolimnas bolina* and *Hypolimnas misippus*, have received most of the attention. *Hypolimnas bolina* and *H. misippus* diverged 8 million years ago (MYa) and share many similarities; both have a pantropical distribution, and the females of both species are polymorphic Batesian mimics of toxic models (Smith 1976; Marsh et al. 1977; Sahoo et al. 2018). In contrast, males are monomorphic and have retained what is likely to be the ancestral phenotype of white-spotted black wings. Nonetheless, the 2 species also differ in many aspects. *Hypolimnas misippus* females are mimics of the 4 morphs of the African Queen, *Danaus chrysippus*, while *H. bolina* females are mimics of several *Euploea* species. *Hypolimnas bolina* has also received special interest for its coevolution with the endosymbiont *Wolbachia* (Dyson et al. 2002; Charlat et al. 2009). In *H. bolina*, *Wolbachia* has a male-killing phenotype that promotes spread of the endosymbiont through females but has counter-evolved a suppressor locus that rescues male butterflies (Hornett et al. 2006).

The diversity in phenotype, precision of mimicry and intricate coevolution, make *Hypolimnas* a remarkable genus for evolutionary biology studies. However, there are few genomic resources to date. Here, we present chromosome-level assemblies for *H. misippus* (HypMis_v2) and *H. bolina* (HypBol_v1) and use our high-quality *H. misippus* assembly containing the Z and W chromosomes to explore the evolution of sex chromosomes in Lepidoptera. First, we compare synteny and TE content between the 2 *Hypolimnas* assemblies. Next, we evaluate the annotation completeness and gene content by comparison to the closely related painted lady butterfly, *Vanessa cardui.* Then, we examine the origin of the W and Z chromosomes across the Lepidoptera by analyzing synteny between *H. misippus* and a diverse set of 10 Lepidoptera species in different ditrysian families. Finally, we investigate the hypothesis of a B chromosome origin of the W chromosome by comparing homology between the W chromosome, autosomes, and Z chromosome within *H. misippus* and with other Lepidoptera species.

## Results

### Genome Assemblies and Synteny between HypMis_v2 and HypBol_v1

The size of the final assemblies was 438.07 Mb for HypMis_v2 and 444.68 Mb for HypBol_v1, assembled into 218 and 59 scaffolds, respectively. HypMis_v2 was sequenced using a trio binning strategy, which, by using a combination of short- and long-read sequencing of the parents and offspring of a cross, allows for the assembly of 2 parental haplotypes (Yen et al. 2020). We assembled both parental haplotypes to a quasi-chromosome level, identified the Z chromosome in the paternal (haplotype 1) assembly and included it in the maternal (haplotype 2) assembly, and then scaffolded the maternal haplotype including the Z using Hi-C data. Hereafter, all mentions of the *H. misippus* assembly, HypMis_v2, refer to the Hi-C scaffolded haplotype 2 assembly.

**Fig. 2.**
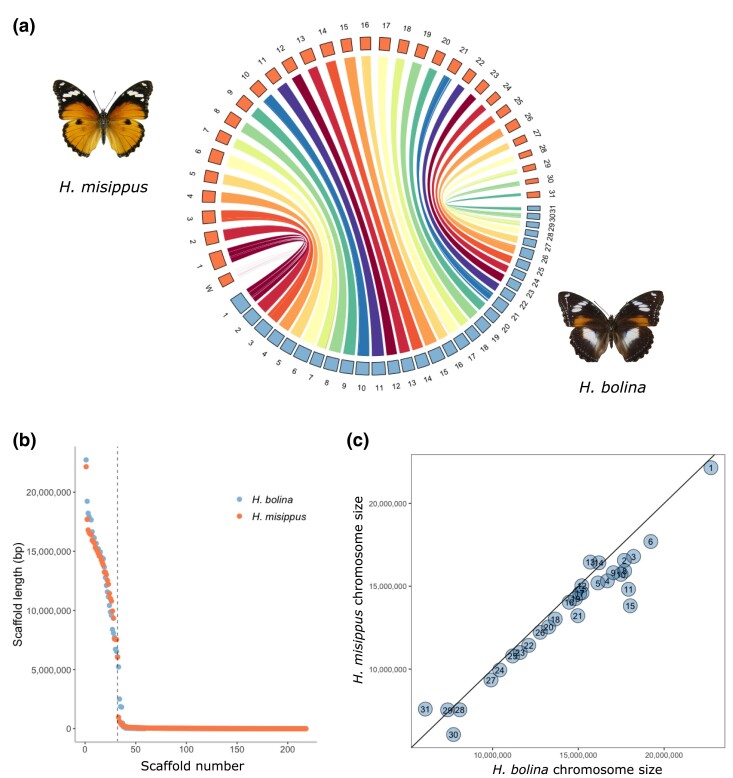
Chromosome-level assemblies for *H. misippus* and *H. bolina.* a) Chromosomal synteny is conserved in both species. b) Chromosome-level scaffolds have been assembled for both species. c) In general, *H. bolina* chromosomes are larger. Chromosome 1 refers to the Z chromosome. *Hypolimnas bolina* image modified from MCZ Harvard University (2023) under CC BY-NC-SA 3.0.

HypMis_v2 was assembled into 32 chromosome-level scaffolds (>6 Mb; 30 autosomes and the Z and W chromosomes) and 186 unplaced scaffolds smaller than 1 Mb (Fig. 2b) and has an N50 of 14.6 Mb, while HypBol_v1 was assembled into 59 scaffolds all placed onto 31 chromosomes, with an N50 of 15.2 Mb. Thus, both species have retained the ancestral karyotype of 31 chromosomes. This karyotype is present in other Nymphalinae species such as the painted lady *V. cardui*, which has a comparable genome size (424.8 Mb) to the 2 *Hypolimnas* species. In general, all chromosomes were slightly larger in HypBol_v1 compared to HypMis_v2 with only 4 exceptions (chromosomes 13, 14, 28, and 31; Fig. 2c). When aligning the 2 *Hypolimnas* assemblies, there were no fusions or fissions among chromosomes, but multiple large rearrangements were observed (Fig. 2a; supplementary fig. S1, Supplementary Material online). The only caveat to these conclusions is that HypMis_v2 was used to scaffold HypBol_v1 before using the linkage map, which could theoretically have caused us to miss differences in the *H. bolina* chromosomal structure. However, the linkage map had 31 linkage groups, providing an independent line of evidence that the 2 species have the same karyotype. Moreover, there were no conflicts between the linkage map and the assembly curated with ragout, and therefore, no evidence of interchromosomal rearrangements (e.g. translocations) between the species. Finally, 12 large inversions on multiple chromosomes are apparent in the alignment of the 2 assemblies (supplementary fig. S1, Supplementary Material online).

### TEs and Repeat Content

**Fig. 3.**
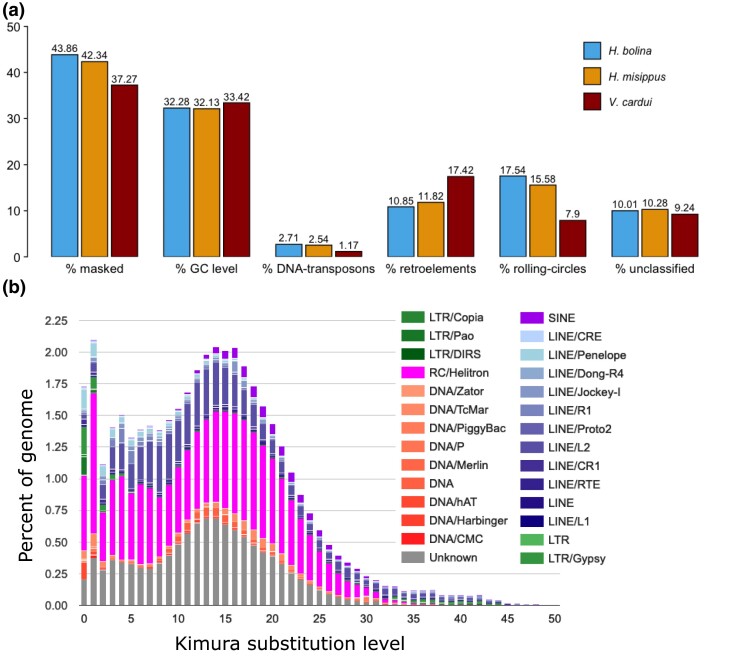
a) Repeat content of the *H. bolina*, *H. misippus*, and *V. cardui* assemblies. b) Repeat landscape of the *H. misippus* assembly. Helitrons and long terminal repeat retrotransposons have undergone a recent expansion, as seen in the higher percentage of the genome covered by *Helitrons*.

In total, the 2 *Hypolimnas* genomes contain similar levels of repeats, 43.86% for HypBol_v1 and 42.34% for HypMis_v2, which are slightly higher than in the painted lady *V. cardui* (37.27%) of comparable genome size (424.8 Mb). The composition of repeats is similar in the 2 *Hypolimnas* genomes, while they differ substantially from the painted lady (Fig. 3). The contribution of rolling circles (also known as *Helitrons*) and DNA transposons is more than double in *Hypolimnas* than in the painted lady, while retroelements are >1.5-fold higher in the painted lady (Fig. 3). This suggests a shift in TE activity, with *Helitrons* and DNA transposons playing a more important role in *Hypolimnas* species. In both *Hypolimnas*, there has been both a recent and a more ancient expansion of *Helitron* family TEs (Fig. 3b). Finally, the percentage of unclassified repeats and GC content is broadly the same in the 3 species.

### Gene Content and Completeness of the Annotation

In total, 19,721 genes were annotated in HypBol_v1 and 21,784 coding mRNAs including all isoforms of the same gene, while for HypMis_v2, 20,293 genes and 22,468 coding mRNAs were predicted (supplementary table S1, Supplementary Material online). These numbers are higher than other Nymphalidae species such as the painted lady butterfly (*V. cardui)*, whose latest annotation (v2.1) includes 13,223 genes and 19,836 mRNAs, with the number of genes being considerably larger in the 2 *Hypolimnas* species (supplementary fig. S2 and table S1, Supplementary Material online). Analysis with BUSCO using the *insecta_odb10* benchmarking set showed that the completeness of the genome and annotation were 98.7% and 98.5% for HypMis_v2 and 94% and 92.4% for HypBol_v1 (Table 1). These scores are comparable to other published Nymphalidae assemblies and annotations such as that of *D. chrysippus* (Singh et al. 2022) or the small tortoiseshell butterfly, *Aglais urticae* (Bishop et al. 2021), with those for *HypBol_v1* slightly lower possibly due to errors introduced by Nanopore sequencing, which has a high per base error rate.

**Table 1:** BUSCO scores for the genome assemblies and gene annotations of *H. misippus* and *HypBol_v1* calculated using the Insecta_odb10 (*n* = 1367) set of genes

	Species	Complete	Single	Dupl.	Frag.	Miss.
*Genomes*	*H. misippus* haplotype 1 (parental)	98.7	98.5	0.2	0.4	0.9
	*H. misippus* haplotype 2 (maternal)	98.7	98.5	0.2	0.4	0.9
	*H. bolina*	94.0	93.6	0.4	1.8	4.2
*Annotations*	*H. misippus*	98.5	98.1	0.4	0.7	0.8
	*H. bolina*	92.4	91.7	0.8	2.9	4.7

To investigate the possible reason behind the large number of annotated genes in the 2 assemblies, we first evaluated the number of annotated genes in public assemblies given their genome sizes and concluded that, albeit large, the number of genes in HypBol_v1 and HypMis_v2 is not an extreme outlier (Supplementary Information). This large number of genes annotated in the 2 assemblies could be due to a mismanagement of TE masking during the annotation process and a consequent inclusion of TE genes in the annotation. To evaluate if this was the case and produce a cleaner annotation, we scanned the annotations to look for known protein sequences and domains. We found that 631 and 1,403 repeat related genes in the HypBol_v1 and HypMis_v2 annotations, respectively (supplementary table S2, Supplementary Material online, and Supplementary Information). With this, we produced 2 lists of 15,858 and 15,523 proteins unrelated to TEs and repeats and with known protein domains for HypMis_v2 and HypBol_v1, respectively (Supplementary Information), which is the expected number of genes for genomes of their sizes (supplementary fig. S3, Supplementary Material online).

Despite having a larger number of genes and mRNAs, the 2 *Hypolimnas* annotations show a smaller total number of exons and introns than *V. cardui* (supplementary fig. S2, Supplementary Material online). This is because the 2 *Hypolimnas* annotations have more single exon genes and their mRNAs have, on average, a smaller number of exons, which might be a difference produced by the distinct annotation pipelines. The total length of mRNAs and genes in the 2 *Hypolimnas* is shorter than in *V. cardui*, which results in a smaller percentage of the genome covered by them. However, this trend is different for exons, which are on average the same length in the 3 species, have a comparable total length, and cover a similar percentage of the genome. Thus, the longer total length of mRNAs and genes in *V. cardui* is due to a total longer length of introns, due to a higher number of introns per mRNA and an average longer length.

### Confirmation of the Identity of the W Chromosome of *H. misippus*

The HypMis_v2 assembly contains an assembled putative W chromosome. To confirm its identity, we performed 4 analyses. First, we calculated read depth for the putative Z, W, and autosomes from the trio parents. As expected given that females are ZW and males are ZZ, the W and Z of the mother show roughly half the depth as the autosomes, while in the father, the Z shows the same depth as the autosomes (supplementary table S3 and fig. S4, Supplementary Material online). Although some reads map to the putative W in the father, it presents very low depth—these are probably due to mismapping because of the high repeat content. Second, we aligned HypMis_v2 to complete assemblies (autosomes with Z and W) of closely related species *V. cardui* and *Junonia coenia* (Supplementary Information). With this, we identified correspondence between chromosomes based on their similarity with the putative W chromosome of *H. misippus* sharing the most similarity with the W chromosome of *V. cardui* and *J. coenia.* Third, we evaluated GC content, as unusually high GC content appears to be a common feature of lepidopteran W chromosomes (Wan et al. 2019; Lewis et al. 2021; Lohse et al. 2021; Berner et al. 2023), pattern that the putative W exhibits (supplementary fig. S5, Supplementary Material online). Fourth, we evaluated repeat content, as W chromosomes tend to contain more repeats. The putative W chromosome of *H. misippus* is the chromosome with the highest repeat content and the highest percentage of repeats and contains a much higher percentage than what would be expected given its length (supplementary figs. S6 and S7, Supplementary Material online).

### Comparisons of W Chromosomes Reveals a Possible Single Common Origin in Ditrysian Lineages

**Fig. 4.**
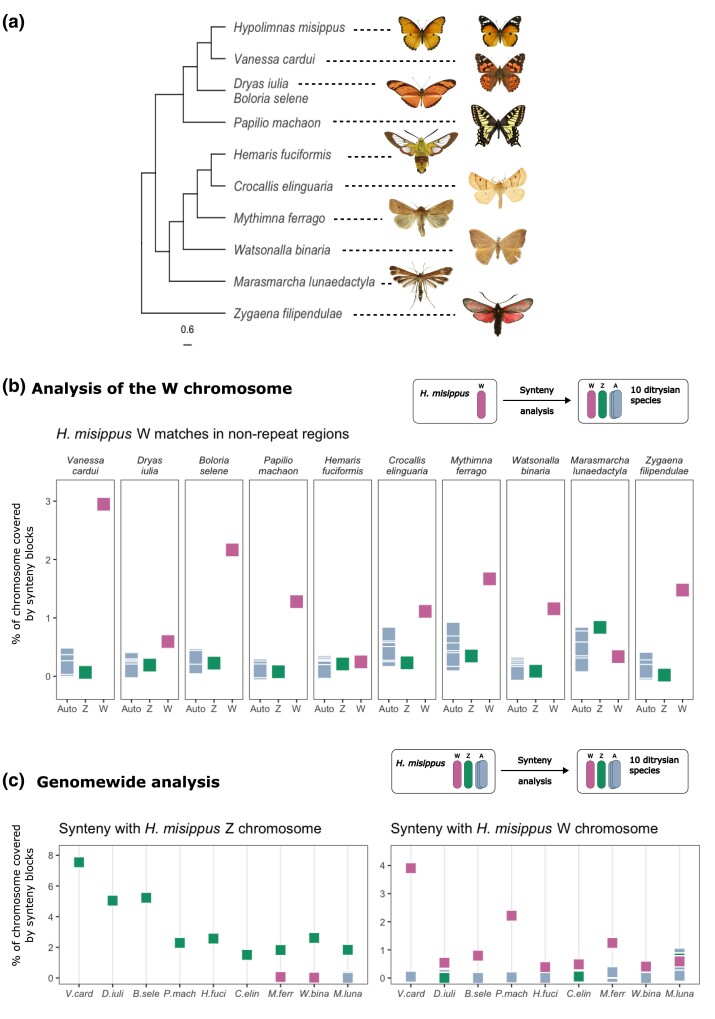
Genome-wide comparisons suggest a single origin of ditrysian W chromosomes and show deep conservation of the Z chromosome. a) Phylogenetic tree modified from Kawahara et al. (2019) including the species used in the synteny analysis. b) Percentage of query *H. misippus* W chromosome covered by synteny blocks of the 32 chromosomes of 10 ditrysian species. W *H. misippus* chromosome used as query (a schematic of the method is shown on top). c) Subset results of genome-wide analysis. Percentage of Z (left) and W (right) chromosome covered by synteny blocks to other ditrysian species. W chromosome, Z, and autosomes are shown. A schematic of the method is shown on top. Species names that have been abbreviated: *Vanessa cardui*, *Dryas iulia*, *Boloria selene*, *Papilio machaon*, *Hemaris fuciformis*, *Crocallis elinguaria*, *Mythimna farrago*, *Watsonalla binaria*, *Marasmarcha lunaedactyla*, and *Zygaena filipendulae.*

Using our HypMis_v2 assembly, we set out to explore the evolutionary origins of the W chromosome in Lepidoptera. First, we wanted to clarify whether the W has a single origin in the Lepidoptera or evolved independently multiple times (Fig. 1d and e). To explore the evolution of W chromosomes within ditrysian lineages, we compared the *H. misippus* W chromosome to a diverse set of 10 Lepidoptera species, including *V. cardui*, *Hemaris fuciformis*, *Mythimna farrago*, *Marasmarcha lunaedactyla*, *Dryas iulia*, *Boloria selene*, *Papilio machaon*, *Crocallis elinguaria*, *Watsonalla binaria*, and *Zygaena filipendulae* (Fig. 4a). These species were chosen as they have a publicly available high-quality assembly containing a W chromosome (supplementary fig. S8, Supplementary Material online) and cover distinct ditrysian lineages, including 8 distinct superfamilies: *Zyganoidea*, *Papilionoidea*, *Pterophonoidea*, *Pyraloidea*, *Drepanoidea*, *Noctuoidea*, *Geometroidea*, and *Bombycoidea*. To evaluate the origin of the W chromosome, we applied an approach that uses syntenic blocks search to infer genetic homology. First, we compared the W chromosome of *H. misippus* to the genomes of other Lepidoptera. If W chromosomes across the Lepidoptera share a common origin, we expect them to share more synteny blocks with each other than with any autosome or the Z chromosome. We searched for syntenic blocks between the *H. misippus* W chromosome and all the chromosomes of each target species (including the W) and found that there is a high proportion of syntenic blocks among W chromosomes (Fig. 4b). The highest degree of sharing is seen between W chromosomes of closely related species such as *H. misippus* and *V. cardui* (45 MYa), but is also true even for highly divergent lineages such as *Z. filipendulae*, which diverged from *H. misippus* 156 MYa (Kawahara et al. 2019). Interestingly, there is no consistent pattern in the distribution of syntenic blocks along the *H. misippus* W chromosome (supplementary fig. S9, Supplementary Material online), which might suggest that different regions show homology with the *H. misippus* W chromosome across species and chromosome types (W, Z, and autosomes).

We then performed the same analysis including all chromosomes of *H. misippus* as the query. With this, we could test for homology between all autosomes and sex chromosomes of *H. misippus* with each chromosome of the target species. Unlike the above method, this approach produces only primary matches for each query chromosome and is thus efficient at finding homologous pairs. This approach yielded the same results as the former, in which the W chromosome of *H. misippus* shares the greatest proportion of syntenic blocks with the W of most species, with *M. lunaedactyla* being the one exception (Fig. 4c, right). Finally, the percentage of the Z chromosome covered by synteny blocks was the highest with other Z chromosomes in all species comparisons (Fig. 4c, left), suggesting that it is highly conserved.

### A Multispecies Comparison Reinforces the Hypothesis of the Single Origin of the W Chromosome and Reveals a Neo-Z Chromosome

In light of the above results suggesting a single origin of the W chromosome in ditrysian lineages, we decided to explore this further by comparing the analyzed ditrysian assemblies with each other. We performed pairwise searches for syntenic blocks between species pairs and observed consistent results. First, the Z chromosome is deeply conserved in multiple species pairs and the proportion of syntenic blocks between Z chromosomes of different species decays with species divergence in a similar manner to autosomes (supplementary figs. S10 and S11, Supplementary Material online). Second, the W chromosome shows a more variable pattern of synteny between species but shows evidence for conservation even between distant species pairs (supplementary fig. S11, Supplementary Material online). Nonetheless, several of the comparisons found no syntenic blocks between W chromosomes, again indicating that the syntenic blocks shared with the *H. misippus* W differed between target species.

Finally, the cross-species comparison revealed that the Z chromosome of *M. lunaedactyla* (chromosome name OV181339.1; identity as the Z chromosome assigned in the public assembly) presented high levels of synteny with chromosome 19 of most other species (supplementary figs. S11 and S12, Supplementary Material online). This could suggest a historical fusion event between the ancestral chromosome 19 and the Z chromosome of *M. lunaedactyla* creating a neo-Z chromosome. The pattern of syntenic blocks seen in the OV181339.1 chromosome was similar for all species compared, with approximately half of the chromosome showing homology to chromosome 19 and half to the Z chromosome (supplementary fig. S13, Supplementary Material online). Furthermore, the pattern of synteny with other species showed that translocations between the parts belonging to the ancestral Z and ancestral chromosome 19 have resulted in an interleaved pattern of homology. Consistent with these results, BUSCO matches of chromosome OV181339.1 are shared with chromosome 19 and chromosome 1 (the Z chromosome) of *Melitaea cinxia* (supplementary figs. S11 and S12, Supplementary Material online).

### Ambiguous Evidence for the Origin of W Chromosomes from the Same Autosome Pair as the Z

**Fig. 5.**
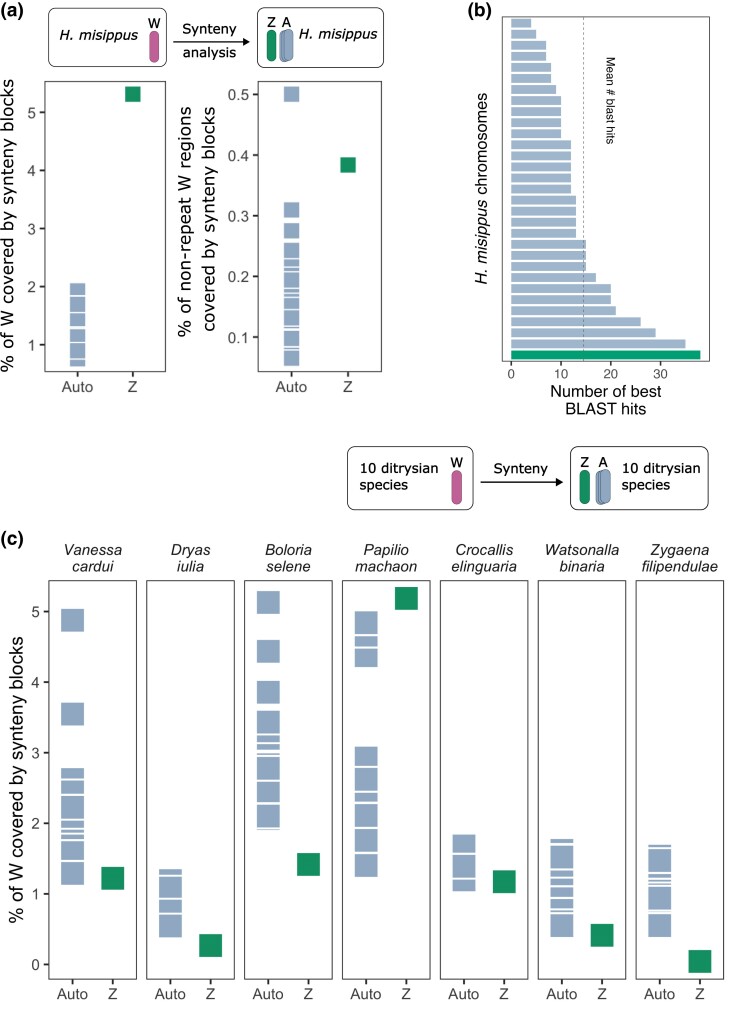
Genomic synteny of the *H. misippus* W chromosome provides ambiguous and weak evidence on its origin. a) Synteny analysis of the *H. misippus* W chromosome and the autosomes and Z chromosome shows that the W and Z chromosomes have the highest similarity. The analysis of nonrepeat regions shows that the Z chromosome is the second most similar chromosome to the W after chromosome 19 (right). A schematic of the method is shown on top. b) Number of best BLAST hits of the proteins found in the *H. misippus* W chromosome to the other chromosomes. Numbers summarized by chromosome with the Z highlighted. c) Synteny analysis of the W chromosomes of several Lepidoptera genome assemblies compared to the autosomes and Z of the same species. A schematic of the method is shown on top. *Hemaris fuciformis* image modified from Didier Descouens under CC BY-SA 4.0. *Dryas iulia* image modified from Moore (2023) under CC-BY. *Watsonalla binaria* image modified from Lennuk (2023) under CC BY-SA 4.0.

The second question we wanted to clarify is whether the W chromosome had originated from a B chromosome or from the same autosomal pair as the Z (Fig. 1a and b). To specifically test the hypothesis that the Z and W have a common autosomal origin, we searched for syntenic blocks between the *H. misippus* W chromosome and its remaining 31 chromosomes. We would expect that if the W and the Z share a common autosomal origin, they might share tracts of homologous sequence that date back to their common ancestor. As such, we might expect that the Z and W would each share more syntenic blocks with each other than with any of the autosomes. We found that the Z chromosome indeed shares a higher proportion of nonoverlapping syntenic blocks with the W (Fig. 5a). However, the W chromosome is the chromosome with the highest percentage of repeat regions (71% in *H. misippus*), which could interfere with the analysis (i.e. if the sex chromosomes share many common repetitive sequences that may have accumulated independently on each sex chromosome). Furthermore, the Z chromosome is the longest chromosome in *H. misippus* and has the highest absolute length of repeats (supplementary figs. S4 and S5, Supplementary Material online). To take this into account, we excluded all matches found in repetitive regions. With this correction, the Z chromosome shared the second highest proportion of syntenic blocks with the W after chromosome 2.

To further explore the possible homology between the W and Z chromosomes, we extracted the predicted amino acid sequences of 885 annotated proteins in the *H. misippus* W chromosome that are unrelated to TEs and repeats and contain known protein domains and searched for homology in the autosomes and Z chromosomes using BLASTp. We found that the Z chromosome is placed 1st in the number of best BLAST protein hits with 38 proteins mapping to it, closely followed by chromosome 2 with 35 proteins mapping to it (Fig. 5b). The average number of best BLAST hits was 15.

In view of the differences between these results and previously published homology tests in other species, we decided to expand the analysis to include other Lepidoptera species. We searched for syntenic blocks between the W and the other 31 chromosomes in 6 of the taxa used in the previous analysis (*D. iulia*, *B. selene*, *P. machaon*, *C. elinguaria*, *W. binaria*, and *Z. filipendulae*) that cover distinct ditrysian lineages (including 4 superfamilies: *Zyganoidea*, *Papilionoidea*, *Drepanoidea*, and *Geometroidea*). A low percentage of the W chromosome was covered by synteny blocks when comparing it to the Z in any species (suggesting no homology) except for *P. machaon*, which contrasts with the results in *H. misippus* (Fig. 5c). However, these analyses were performed using unmasked genomes and including all regions, which could be interfering with the results.

## Discussion

*Hypolimnas* species have become a focus for studies of evolutionary genetics, due to their mimetic coloration and coevolution with *Wolbachia* parasites. Here, we have assembled chromosome-level reference genomes for *H. misippus* and *H. bolina* and produced RNA-informed annotations for both genomes. HypBol_v1 represents the first assembly available for *H. bolina*, while HypMis_v2 is a significant improvement on a previously published genome, with higher contiguity and higher N50 and BUSCO scores. Furthermore, the annotation is also improved as we used RNA-seq data from *H. misippus* to inform our annotation, as opposed to only homology methods. We present these 2 useful resources and use the *H. misippus* assembly to shed light on the evolutionary origins of W chromosomes in Lepidoptera.

Several models have been proposed to explain the origin and evolution of the W chromosome in Lepidoptera (Lukhtanov 2000; Sahara et al. 2012; Fraïsse et al. 2017; Dai et al. 2022; Berner et al. 2023). The deep conservation of the Z chromosome and its apparent lack of homology with the W have been suggested to be evidence of a B chromosome origin of the W chromosome (Fraïsse et al. 2017). Outside the Lepidoptera, it has been commonly found that heteromorphic sex chromosomes retain a degree of similarity, evidence of their shared autosomal origin (Wright et al. 2016). Thus, if the W chromosome originated from the same homologous autosome pair as the Z, we might expect to find some residual sequence similarity between the W and Z, as some recent evidence seems to suggest. However, opposing evidence has also been presented, suggesting a lack of similarity between W and Z chromosomes which could point to the evolution of the W from a B chromosome (Fraïsse et al. 2017; Lewis et al. 2021). Furthermore, the lack of similarity among W chromosomes of Lepidoptera species has led to the suggestion of multiple independent recruitments from B chromosomes (Lewis et al. 2021; Dai et al. 2022). Taking this into account, we set out to address 2 questions. First, do W chromosomes in Lepidoptera share a common origin, that is, did they evolve once or multiple times independently? Second, did the W chromosome evolve from the same homologous pair of autosomes as the Z or from a B chromosome? Our assembly of the W chromosome of HypMis_v2 is an ideal system to explore these questions, as its large size (13.23 Mb) compared to other W assemblies (e.g. 1.87 Mb in *Z. filipendulae* or 2.1 in *D. iulia*) increases our chances of detecting homology even in scenarios of extensive degradation.

Using tests for homology between the W and remaining 31 chromosomes in *H. misippus* and other Lepidoptera, we show that there is evidence for a single common origin of all W chromosomes across the Lepidoptera. This contrasts with previous in which the lack of similarity among W chromosomes of Lepidoptera species was suggested to be the result of multiple independent recruitments from B chromosomes (Lewis et al. 2021). Similarly, phylogenies of W genes in the Asian corn borer could suggest multiple independent origins of the W chromosome from the same autosomal pair as the Z (Dai et al. 2022). Regardless of the specific event that led to the formation of the W chromosome (i.e. from a B chromosome or from an autosome), if the W chromosomes of ditrysian lineages share a common origin, we would expect them to share homologous sequence tracts that date back to their common ancestral chromosome, and therefore, we expected to find more syntenic blocks between W chromosomes than between W and Z or W and autosomes. Comparing the *H. misippus* W chromosome to the genomes of 10 other ditrysian species, our findings matched this expectation in all but one species. These results suggest that W chromosomes of ditrysian lineages may share a single common origin, which contrasts with evidence of rapid turnover of W chromosomes in Lepidoptera (Lewis et al. 2021; Dai et al. 2022). However, it is possible that some species have retained the ancestral W, while some other species have recruited other B chromosomes as suggested. For example, in our test for homology between the *H. misippus* W chromosome and each of the chromosomes of *M. lunaedactyla*, the W chromosome of *M. lunaedactyla* did not stand out above the Z and some autosomes. Our results also show unambiguous evidence for homology among Z chromosomes of the studied ditrysian species, which is consistent with evidence suggesting deep conservation of the Z chromosome across the Lepidoptera (Fraïsse et al. 2017).

Secondly, we show that there is some evidence for homology between the W and the Z chromosome, but that our results are ambiguous. While the synteny results are weak toward any hypothesis, the BLAST results support a common origin of the Z and W. This contrasts with the homology tests performed in *Danaus plexippus* (Lewis et al. 2021), *Kallima inachus* (Yang et al. 2020), and *D. iulia* (Lewis et al. 2021), where the evidence was inconsistent with a common origin of the W and the Z chromosome and is more in line with the results in the Asian corn borer, *Ostrinia furnacalis*, a moth belonging to the Pyraloidea, and *Pieris mannii* which suggest a common autosomal origin of the Z and the W chromosome (Dai et al. 2022; Berner et al. 2023). Our results based on protein-coding genes hint at a possible common origin of the Z and W, but are not consistent with the synteny results which are ambiguous. The low proportion of synteny blocks between the W and the Z in 5 of the 7 species studied could suggest a B chromosome origin or could be the result of the rapid evolution and degradation of the W, which could impede the detection of ancient homology (Vítková et al. 2007; Yoshido et al. 2013). Taken together, the homology tests between the W and remaining 31 chromosomes in *H. misippus* and other Lepidoptera provide weak and ambiguous evidence for the origin of the W from the same homologous pair of autosomes as the Z. Alternatively, our finding of synteny blocks between the *H. misippus* W and the Z could point to a fusion of the Z chromosome with an autosome and subsequent formation of the W (Fig. 1c). This hypothesis would fit the mounting evidence supporting a Z0 system in nonditrysian Lepidoptera and early-diverging ditrysian lineages (Lukhtanov 2000; Sahara et al. 2012; Fraïsse et al. 2017; Hejníčková et al. 2019).

Overall, we present evidence of a single common origin of W chromosomes in the Lepidoptera. Furthermore, our results are ambiguous on a possible origin from the same autosomal pair as the Z or perhaps to a Z autosome fusion event that culminated with the formation of the W. Crucially, the rapid evolution of the W chromosome could cause the lack of similarity observed between Z and W chromosomes in some species and hinders the elucidation of its origin. Further studies including more species across the Lepidoptera are necessary to clarify whether the W chromosome evolved from an autosome or from a B chromosome and to shed light on the possible shared origin of W chromosomes, particularly including nonditrysian lineages.

## Materials and Methods

### *Hypolimnas misippus* Butterfly Rearing and Cross Preparation

A trio binning approach was used for sequencing of the *H. misippus* assembly, which consists of sequencing the parents and offspring from one family. First, butterflies were obtained from Stratford-upon-Avon Butterfly Farm, UK, and reared in greenhouses in Madingley, Cambridge, UK. Larvae were fed *Portulaca oleracea* and *Portulaca quadrifida*. Adult butterflies were kept in a large cage (1.5 m × 1.5 m × 2 m) and observed until mating occurred when mating pairs were transferred to separate smaller cages during copulation. One mated pair was used for trio-based genome sequencing, and their offspring were reared until pupation and then flash frozen as pupae in liquid nitrogen.

### *Hypolimnas misippus* Trio Binning Genome Assembly

One *H. misippus* family was used for sequencing and trio binning genome assembly. DNA was extracted using MagAttract HMW DNA Kit (QIAGEN). The 2 parents were sequenced using Illumina short-read sequencing, which resulted in 368.67 and 346.56 million read pairs and a total yield of 55.67 and 52.33 Gb of data from the mother and father, respectively. One of the offspring was sequenced using PacBio HiFi long-read sequencing (total yield of 13.46 Gb of data and N50 of 13,493). Trio binning enables the independent assembly of the 2 parental haplotypes. First, yak-r55 (Li 2022) was used to create a kmer database from the parental Illumina data. Then, hifiasm-0.7-r256 (Cheng et al. 2021) was run in trio mode to assemble the PacBio HiFi long-read data, followed by the purging of haplotypes and overlaps using purgedups v1.2.3 (Guan 2022). Haplotype 1 corresponds to the paternal haplotype, while haplotype 2 corresponds to the maternal haplotype. The Z chromosome found in haplotype 1 was identified using differences in read coverage between sexes and included in haplotype 2. With that, haplotype 2 becomes a complete assembly containing both Z and W chromosomes. All subsequent analyses were performed using haplotype 2.

### Curation of HypMis_v2 with Hi-C

To place the assembled scaffolds into chromosomes, Hi-C sequencing was used, which is a chromosome conformation capture technology that provides information about the 3D interactions between genomic loci. An offspring from an unrelated mating was flash frozen in liquid nitrogen and used for Hi-C sequencing and analysis. A total of 386.99 million read pairs were produced, which represented 58.45 Gb of Hi-C data. Cram files were converted to fastq (2 files, one for each read pair end. The subsequent processing to produce the *H. misippus* assembly was done with Juicer v1.6 (Durand et al. 2016b). Juicer transforms raw Hi-C data into a list of contacts, which defines pairs of genomic positions that were in close physical contact during the experiment. Then, the main reference assembly was curated using the 3D-DNA pipeline, which corrects misassembles, anchors, and orders and orients fragments of DNA based on the Hi-C data (Durand et al. 2016a; Dudchenko et al. 2017). 3D-DNA generated assembly heatmaps as part of its workflow, which indicate the frequency of contact between pairs of genomic locations. Obvious errors in the genome assembly such as large genomic inversions were manually edited by examining the Hi-C heatmaps using the Juicebox tool (Durand et al. 2016a; Dudchenko et al. 2018). Finally, the edited assembly was exported as an *.assembly* file and converted to a final fasta assembly file using the “run-asm-pipeline-post-review.sh” script setting –editor-repeat-coverage to 6.

### HypBol_v1 Genome Curation with Ragout

The *H. bolina* reference assembly (HypBol_v1) was produced using a combination of Nanopore long-read sequencing and a linkage map. Pupae were purchased from the Stratford-upon-Avon butterfly farm, which obtains farmed lineages from Southeast (SE) Asia. One female pupa was used for sequencing. High molecular weight DNA was extracted following a phenol chloroform and glass capillary hook protocol following (Quick 2018). A library was prepared for Nanopore sequencing using the Nanopore Ligation Sequencing Kit (SQK-LSK 109) following a LSK109 bead free library preparation protocol (Tyson 2020). Post library preparation, a total of 1.78 µg DNA remained and was sequenced across 2 R9 chemistry Nanopore MinION flow cells, resulting in 7.35 million reads totaling 14.16 Gb. Adapters were removed using Porechop v0.2.4 (Wick 2022) and reads assembled using redbean (Ruan and Li 2020). Assembled contigs were split into bins using MaxBin2 (Wu et al. 2016), and bacterial contigs and reads were removed from the data using blobtools2 (Challis et al. 2020). This produced an assembly with 13,492 contigs and an N50 of 1.4 kb.

Leveraging the high synteny expected from the 2 *Hypolimnas* assemblies, Ragout v2.3 (Kolmogorov et al. 2014, 2018) was used to improve the initial *HypBol_v1* assembly using HypMis_v2 as a reference. First, the 2 genomes were soft-masked using RepeatMasker (Smit et al. 2015), creating the repeat library based on the genome being masked. Then, Cactus (Paten et al. 2011) was used to align both genomes using Python 3.8. The resulting HAL alignment was converted to MAF format using the hal2maf utility from the HAL program (Hickey et al. 2013). Finally, Ragout was run using the MAF alignment between HypBol_v1 and HypMis_v2 as input.

### Rearing of *H. bolina* Individuals

A linkage map was used to improve the HypBol_v1 assembly and place the assembled scaffolds into chromosomes. Two families (178303XX and 182703XX) were reared and sequenced. First, female *H. bolina* purchased from Stratford-upon-Avon Butterfly Farm of SE Asian origin were mated to wild-caught males from Mo’orea (French Polynesia) at the University of California Berkeley Gump Station research facility. Female SE Asian-Mo’orea F1 hybrid offspring were then mated to pure Mo’orea F1 males. The F2 offspring of one of these crosses is family 178303XX. At the same time, male SE Asia-Mo’orea F1 hybrids were mated to pure SE Asia F1 females. The F2 offspring of one of these crosses is family 182703XX. Butterflies were kept in a large outdoor cage for mating under observation. Any copulating pairs were separated into a small cage. The mated female was then placed in an oviposition cup containing a small plant, e.g. *Asystasia* sp., and allowed to lay eggs. Hatched eggs were moved to a rearing box with suitable food plant, e.g. *Ipomoea* sp., and caterpillars reared until pupation. Pupae were moved to individual cups for emergence; adults left to dry for 1 day and then used for further matings or stored in −80°C freezer.

### Illumina Library Preparation of *H. bolina* Family Samples

The offspring of the *H. bolina* families (F2) were processed to extract the DNA and prepare the libraries for Illumina sequencing. DNA extractions were carried out using a custom protocol using PureLink buffers and homemade magnetic beads following a modified version of the protocol from (Kučka and Frank Chan 2022). The only modification of the protocol is that volumes are halved. Briefly, a small piece of thorax tissue (1/10) is placed in an 8-tube PCR strip. Then, 45 µL of PureLink Digestion buffer and 10 µL of Proteinase K (20 mg/mL) are added, and the mix is incubated at 58°C with shaking (500 rpm) for 2 to 3 h. Afterwards, 2 µL of RNAseA is added (DNAse free, 10 mg/mL) and incubated for 10 min at room temperature. Then, 45 µL of PureLink lysis buffer is added to the mix and incubated at 58°C for 30 min with shaking (500 rpm). Following that, a homemade magnetic bead mix is used to extract the DNA from the lysate. First, 37.5 µL of magnetic beads is added together with 75 µL of lysate to a 96-well plate. After mixing, the samples are incubated for 15 min at room temperature, then the plate is placed on a magnetic stand for 10 min, the supernatant is removed, and the beads are cleaned with 80% ethanol. After drying out, 50 µL of 10 mM Tris (pH = 8) is added to elute the sample and incubated at 45°C for 15 min without resuspending. Then, the beads are resuspended and incubated for 20 min at room temperature. Finally, the plate is placed on the magnetic stand, and after 10 min, the supernatant (the DNA) is transferred to a fresh tube.

The F2 were sequenced using a Nextera-based library preparation at intermediate coverage (∼11X). A secondary purification using magnetic SpeedBeads (Sigma) was performed prior to Nextera-based library preparation. Libraries were prepared following a method based on Nextera DNA Library Prep (Illumina, Inc.) with purified Tn5 transposase (Picelli et al. 2014). PCR extension with an i7-index primer (N701 to N783) and the N501 and N502 i5-index primers was performed to barcode the samples. Library purification and size selection were done using the same homemade beads as above. Pooled libraries were sequenced by Novogene Cambridge, UK. Libraries of the parental samples were prepared and sequenced to ∼20× coverage by Novogene Cambridge, UK.

### *Hypolimnas bolina* Linkage Map Construction and Anchoring of the Genome

A linkage map was produced with Lep-Map3 (Rastas 2017) and then used to improve the *H. bolina* assembly and place the scaffolds onto chromosomes. First, sequences were mapped to the reference genome using bwa-mem (Li 2013). PCR duplicates were marked using the MarkDuplicates from Picard tools. Sorted BAMs were then created using SAMtools (Li et al. 2009) and genotype likelihoods computed. The pedigree of individuals was checked and corrected using IBD (identity-by-descent) with a random subset of 10% of the markers (1,270,024 SNPs) following the IBD pipeline from Lep-Map3. These markers were also used to construct the linkage map. Scaffolds were anchored into chromosomes based on the linkage map using LepAnchor (Rastas 2020).

### HypBol_v1 Polishing with Pilon

After anchoring with the linkage map, 3 iterations of Pilon v1.24 (Walker et al. 2014) in diploid mode were run to correct the draft assembly by correcting bases, filling gaps, and fixing misassemblies. Samples used for the Pilon correction were CAM035727, CAM035728, CAM035186, CAM035187, CAM035188, and CAM035189.

### Repeat Annotation

Once the 2 final assemblies had been produced, they were each assessed for repeat content using a custom repeat library. First, a repeat database was built and the repeats of the 2 finished assemblies modeled using RepeatModeler v. 2.0.2a. Each custom library was then combined with the Lepidoptera library extracted from Dfam (Storer et al. 2021). This merged library was used to soft mask the genome using RepeatMasker v 4.1.0 with the cutoff score set to 250 and skipping the bacterial insertion element check. The resulting soft-masked assemblies were used for gene annotation.

### RNA-seq Sample Preparation

To assist with genome annotation, RNA-seq data were obtained from 4 *H. misippus* individuals (2 adults and 2 pupae) and 17 *H. bolina* individuals (6 adults and 11 pupae). Butterflies were purchased from Stratford-upon-Avon Butterfly Farm and kept at room temperature until dissection. Four tissues were dissected out from *H. misippus* pupae and placed in RNA-later (Sigma), wing discs, thorax, head, and abdomen, while in *H. bolina* pupae, 3 tissues were dissected: wing discs, thorax-head, and abdomen. Only abdomen, head, and thorax samples were dissected from adults of either species. Two pooling strategies were followed: (i) 2 *H. bolina* pupae were pooled by individual, pooling head, thorax, abdomen, and wing discs together and sequenced at high coverage (50 M reads), and (ii) 4 *H. misippus* and 15 *H. bolina* adults and pupae were dissected into tissues and pooled by species and tissue. Each pooled sample, 4 for *H. misippus* and 3 for *H. bolina*, was sequenced to 20 M reads. RNA was extracted using a modified TRIzol protocol using the same protocol as in Brien et al. (2022).

### RNA-seq Mapping and Gene Annotation

First, low-quality ends and adaptors from the RNA-seq data were trimmed using TrimGalore! v 0.5.0 (Krueger 2015). Then, the reads were mapped to the soft-masked genomes using STAR v 2.5.0a (Dobin et al. 2013). Two rounds of mapping (2 pass) were performed, including all the splice junction files in the second round. Then, the resulting mapped reads and the soft-masked genome assembly were used to generate a gene annotation using BRAKER v 2.1.5 (Brůna et al. 2021), running it a second time to add UTR annotations with options addUTR = on and skipAllTraining (Keilwagen et al. 2019). A single isoform per gene was selected and completeness of the annotation assessed using BUSCO v5.2.2 (Simão et al. 2015; Seppey et al. 2019) using the Insecta_odb10 (*n* = 1367) set of genes.

The HypMis_v2 and HypBol_v1 protein annotations were scanned for known protein domains using InterProScan v5.66-98.0 with default settings.

### Homology between Chromosomes

To assign homology among chromosome of *H. misippus* and *H. bolina*, we used similarity to the *M. cinxia* assessed by BUSCO. Lepidopteran_odb10 BUSCO assignment of the *M. cinxia* genome was compared to the lepidopteran_odb10 BUSCO assignment of *HypBol_v2* and *HypBol_v1*. *Melitaea cinxia* present 31 chromosomes, which represents the ancestral state of Ditrysia (Wright et al. 2023). *Melitaea cinxia* belongs to the *Nymphalinae* which is the same subfamily as *Hypolimnas*, and the 2 taxa diverged about 30 MYa (Espeland et al. 2018). Chromosome names in HypBol_v1 and *H. misippus* were assigned by choosing the chromosome sharing the highest BUSCO hits with a *M. cinxia* chromosome.

### Confirmation of W Chromosome Identity

The mother (CAM035932) and father (CAM035079) of the trio reared for genome sequencing were used to confirm the identity of the putative W chromosome. Adapters and low-quality ends were trimmed using TrimGalore! (Krueger 2015). Then, fastq files were mapped to the HypMis_v2 using BWA-mem2 (Vasimuddin et al. 2019) and duplicates marked using MarkDuplicatesSpark from GATK (Van der Auwera and O’Connor 2020). Read depth was calculated using SAMtools v1.15 (Li et al. 2009) and averaged across 200 kb genomic windows. Assembly alignment of *V. cardui* (ilVanCard2.1) (Lohse et al. 2021) and *J. coenia* (Jc_v2 from LepBase) was performed using D-Genies (Cabanettes and Klopp 2018) with Minimap2 v2.26 (Li 2018). GC content was calculated using BBtools (https://sourceforge.net/projects/bbmap/).

### Searches of Synteny Blocks

Synteny of the 2 assemblies was assessed using 2 methods. First, the 2 final genome assemblies were aligned using D-GENIES (Cabanettes and Klopp 2018), which produced a paf file that was used to detect candidate inversions between the 2 genome assemblies (supplementary fig. S1, Supplementary Material online). Second, synteny between the 2 assemblies was evaluated using Satsuma2 Synteny (Grabherr et al. 2010) and a Circos plot generated using the circlize package v 0.4.14 (Gu et al. 2014) in R v 4.1.2 (Fig. 2).

To examine the origin and evolution of the W chromosome in Lepidoptera, first, synteny-based homology between the *H. misippus* W chromosome, the Z chromosome, and the autosomes was evaluated using Satsuma2. Satsuma2 is an aligner of whole-genome assemblies intended to find homology based on sequence similarity. Satsuma2 first maps all genomic windows of the query genome to the target genome with a percentage of identity higher than 45% and then filters those hits based on large scale synteny, that is, it keeps only matches that are concordant with each other. Thus, Satsuma2 is not only aligning genomic windows, but also evaluating synteny between those blocks. Finally, Satsuma2 focuses on regions with a high number of hits, to exhaustively evaluate the region around those hits, a strategy analogous to the battleship game. To evaluate synteny between the W and the remaining 31 chromosomes in *H. misippus*, the W chromosome was used as a query and the 31 assembled chromosomes as targets for Satstuma2. Resulting mapped regions were then filtered to keep only nonoverlapping regions using the package GenomicRanges in R v4.1.2 (Lawrence et al. 2013). Satsuma2 does not require input genome assemblies to be masked. Because of its algorithm and filtering steps, repeat regions mapping to multiple places in the genome have decreased score and may be filtered out. However, to evaluate only the synteny of nonrepeat regions, the RepeatMasker output, which details the coordinates of repeats in the genome, was used to filter out repeat regions of the W chromosome. Finally, the effect of sequence identity was evaluated by performing the analysis with no identity filter and applying a threshold of 70% similarity.

The synteny between the W and the rest of the genome was also evaluated in 6 other Lepidoptera species: *B. selene* (GCA_905231865.2), *C. elinguaria* (GCA_907269065.1), *H. fuciformis* (GCA_907164795.1), *P. machaon* (GCA_912999745.1), *W. binaria* (GCA_929442735.1), *Z. filipendulae* (GCA_907165275.2), and *D. iulia* (GCA_019049465.1). In all cases, the W chromosome was used as the query, results were converted to nonoverlapping regions, and percentage of the W covered by matches was calculated. No filter based on repeats was applied, as no repeat library was available for these species.

After that, Satsuma2 was used to compare the synteny of the *H. misippus* W (used as query) and Z chromosomes to 10 Lepidoptera species, the 6 from the previous analysis and also *H. fuciformis* (GCA_907164795.1), *M. lunaedactyla* (GCA_923062675.1), *V. cardui* (GCF_905220365.1), and *M. farrago* (GCA_910589285.2). Chromosomes of all the species analyzed were renamed by their homology to *M. cinxia* as above. Again, results were converted to nonoverlapping regions, repeat regions of the W chromosome filtered, and percentage of the W chromosome covered by matches calculated. Using only the *H. misippus* W chromosome as query ensures that secondary and tertiary matches are also reported. A more specific analysis was produced by using the *H. misippus* assembly as query to search for synteny in the 10 Lepidoptera target species. Using whole assemblies as input limits the results to only primary matches, pairing all homologous autosomes.

### BLAST of *H. misippus* W Chromosome Protein-Coding Genes

To further explore the degree of homology between the *H. misippus* W chromosome and the autosomes and Z chromosome, protein sequence homology was assessed using BLASTp v2.4.0+ (Kent 2002; Camacho et al. 2009). First, a protein BLAST database was built using the protein sequences contained in the *H. misippus* autosomes and the Z chromosome in the HypMisi_v2 annotation that were identified by InterProScan as carrying known protein domains unrelated to TEs and repeats. Then, protein sequences found in the W chromosome were extracted, TE-related proteins filtered out, and the remaining blasted to the database setting the minimum *e*-value to 1*e*−10 and the maximum number of target sequences to be reported to 5,000. The best match for each protein sequence was selected based on *e*-value score, and in cases where 2 matches had the same value, percentage of identity was used. Finally, genome coordinates were extracted from the HypMisi_v2 annotation.

## Supplementary Material

evae215_Supplementary_Data

## Data Availability

Genome assemblies of *Hypolimnas misippus* are available at NCBI BioProject accessions PRJNA1086912 (primary assembly) and PRJNA1086911 (alternate assembly) and https://tolqc.cog.sanger.ac.uk/durbin/jiggins/Hypolimnas_misippus/. *Hypolimnas bolina* are available at NCBI BioProject accession PRJNA1087268. HiFi PacBio and Illumina raw reads of the *H. misippus* trio binning samples are available at ENA's project PRJNA521321. Supplementary Information and the data underlying this article including the genome annotations are available in https://doi.org/10.5281/zenodo.8172459. Code used for the analyses can be found in https://github.com/annaorteu/Hypolimnas_genome_Wchr_evolution.
