# Supplementary material for: The Hypolimnas misippus Genome Supports a Common Origin of the W Chromosome in Lepidoptera
Source: Genome Biol Evol. 2024 Oct 30;16(10):evae215. doi: 10.1093/gbe/evae215 (PMC11523094; doi:10.1093/gbe/evae215)
Supplement: evae215_Supplementary_Data [file evae215_supplementary_data.docx]

The *Hypolimnas misippus* genome supports a common origin of the W chromosome in Lepidoptera

# Supplementary Material

| Feature | *HypBol_v1* | *HypMisi_v2* | *Vanessa cardui* |
| --- | --- | --- | --- |
| Total length: Gene | 160709320 | 167739620 | 240814091 |
| Total length: mRNA | 206176689 | 222209628 | 522618316 |
| Total length: CDS | 29265705 | 32999245 | 38521385 |
| Total length: Exon | 35299426 | 39816515 | 56622152 |
| Total length: Intron | 170992076 | 182514332 | 465996164 |
| Mean number per mRNA: Exons | 6.3 | 6.4 | 9.5 |
| Mean number per mRNA: Introns | 5.3 | 5.4 | 8.5 |
| Mean length: Gene | 8149 | 8265 | 18211 |
| Mean length: mRNA | 9464 | 9890 | 26346 |
| Mean length: Exon | 258 | 277 | 299 |
| Mean length: Intron | 1489 | 1505 | 2754 |
| Total number: Genes | 19721 | 20293 | 13223 |
| Total number: mRNAs | 21784 | 22468 | 19836 |
| Total number: Exons | 136597 | 143687 | 189042 |
| Total number: Introns | 114813 | 121219 | 169206 |
| Total number: Single exon genes | 3868 | 4891 | 1387 |
| % of genome covered by: Genes | 36.1 | 38.3 | 56.7 |
| % of genome covered by: mRNAs | 36.1 | 38.2 | 54.4 |
| % of genome covered by: Exons | 5.6 | 6.3 | 7.2 |
| % of genome covered by: Introns | 30.5 | 32 | 47.1 |

**Supplementary Table 1.** Annotation statistics of *Hypolimnas misippus (HypMisi_v2)* and *H. bolina (HypBol_v1)* compared to *Vanessa cardui.*

| Chromosome | TE gene count |
| --- | --- |
| **2** | 22 |
| **3** | 18 |
| **4** | 16 |
| **5** | 20 |
| **6** | 21 |
| **7** | 14 |
| **8** | 18 |
| **9** | 18 |
| **10** | 21 |
| **11** | 20 |
| **12** | 9 |
| **13** | 21 |
| **14** | 21 |
| **15** | 13 |
| **16** | 16 |
| **17** | 12 |
| **18** | 9 |
| **19** | 21 |
| **20** | 16 |
| **21** | 13 |
| **22** | 15 |
| **23** | 9 |
| **24** | 10 |
| **25** | 13 |
| **26** | 42 |
| **27** | 10 |
| **28** | 8 |
| **29** | 64 |
| **30** | 10 |
| **31** | 11 |
| **W** | 309 |
| **Z** | 31 |
| **Unplaced scaffolds** | 112 |

**Supplementary Table 2.** Number of transposable element related protein coding genes identified in the HypMis_v2 annotation using InterProScan summarised by chromosome.

| Individual | Trio father | Trio mother |
| --- | --- | --- |
| CAMID | CAM035079 | CAM035932 |
| ENA ID | ERR5163381 | ERR5163382 |
| Autosome | 119 | 126 |
| W | 16.6 | 82.5 |
| Z | 118 | 63.5 |

**Supplementary Table 3.** Average read depth in 200kb windows for the mother and father of the trio reared for genome sequencing of *H. misippus.* Average read depth for the Z chromosome of the father is the same as for autosomes, while it is minimal for the W and probably only due to the high repeat content. Average read depth for the Z chromosome of the mother is about half of that of the autosomes, and a similar situation is seen for the W, albeit slightly higher.


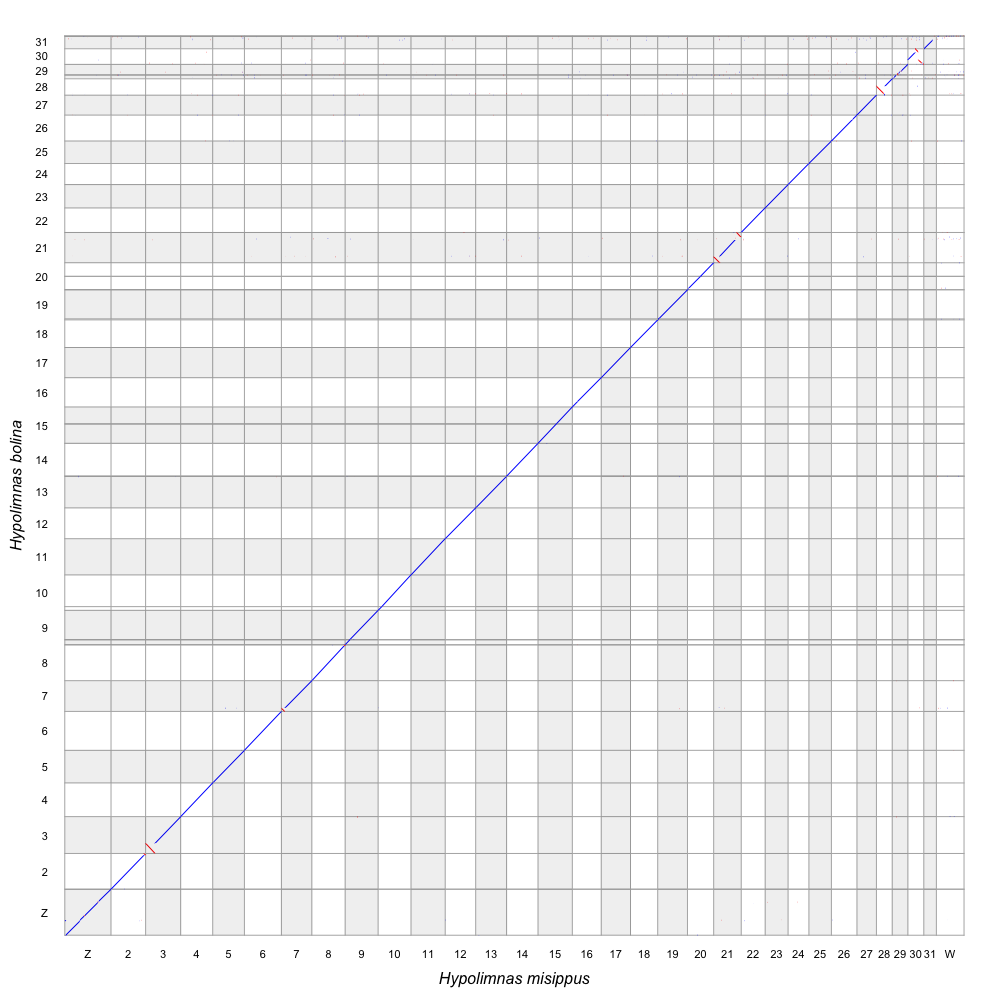


**Supplementary Figure 1.** The D-Genies whole assembly alignment of *Hypolimnas misippus* and *H. bolina* reveals 12 inversions between the two genomes (shown in red).

**
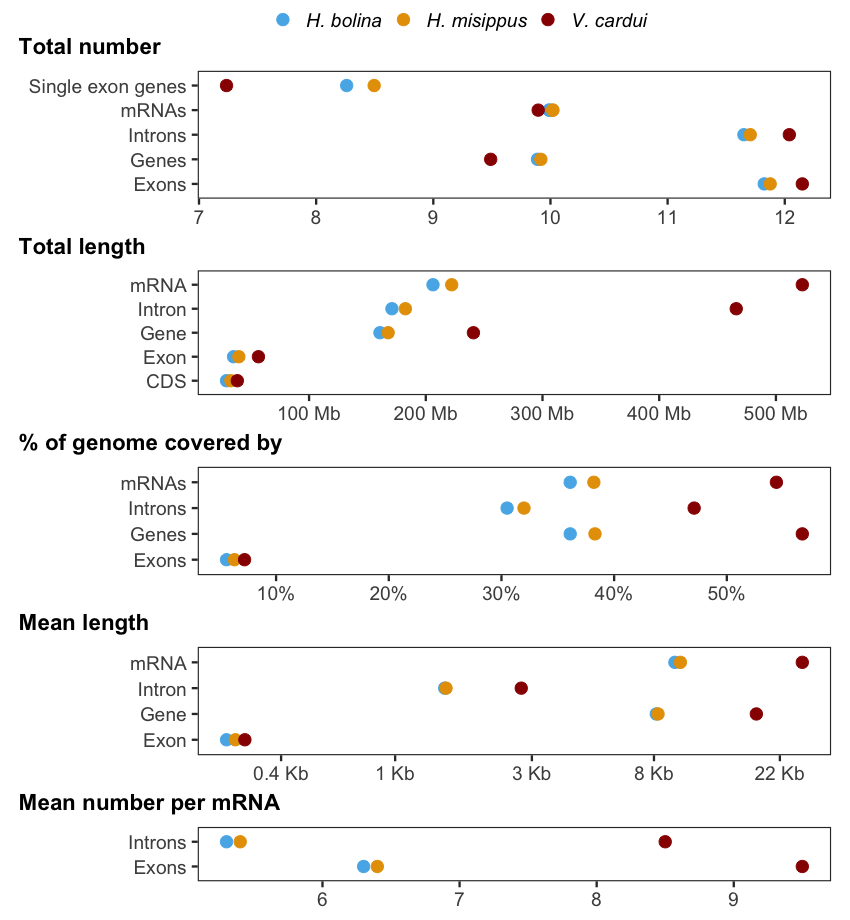
**

**Supplementary Figure 2.** Annotation comparison of *Hypolimnas misippus, H. bolina* and *Vanessa cardui.* For the statistics, pre-mRNA, that is mRNA including exons and introns, have been used. Numbers are found in Supplementary Table 1.


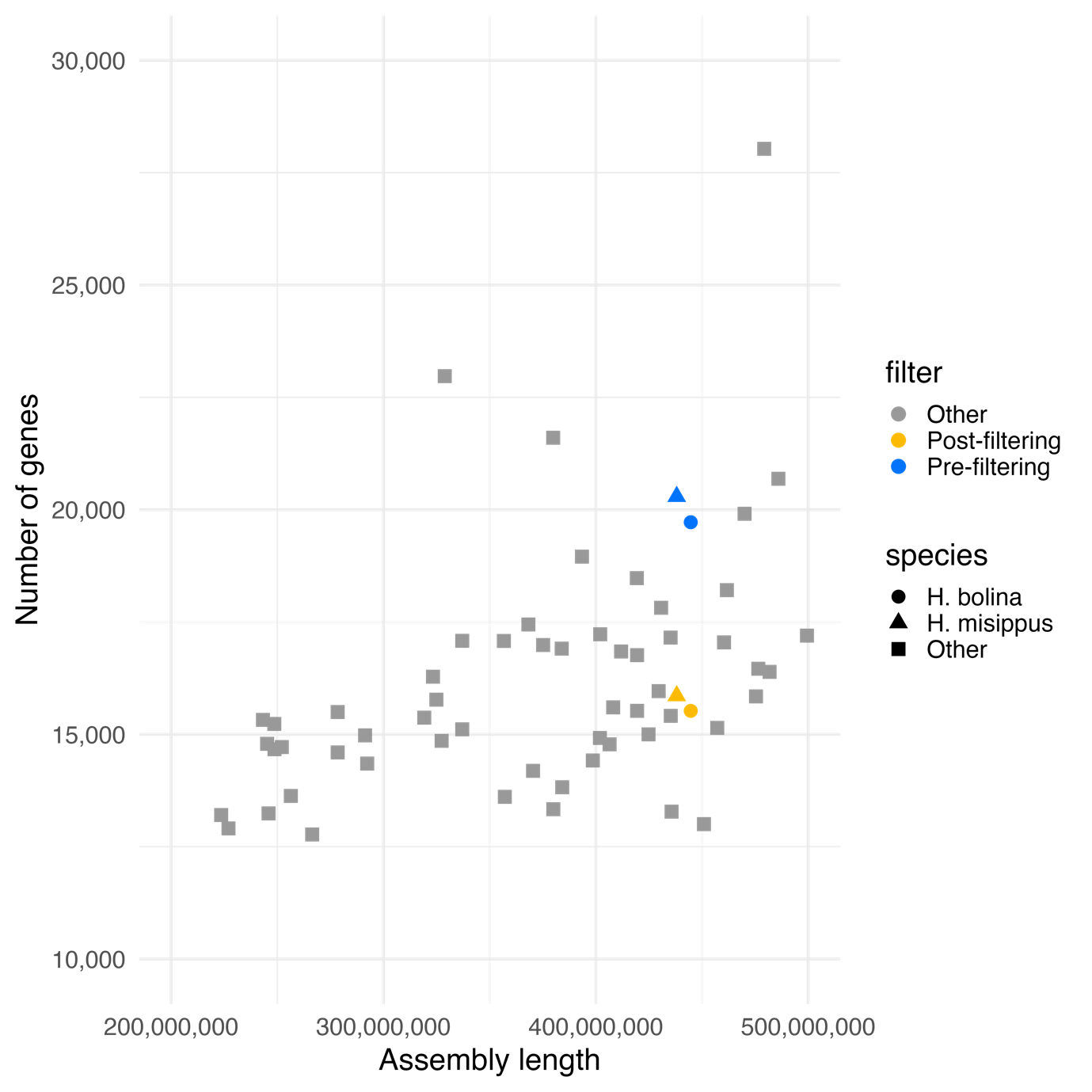


**Supplementary Figure 3.** Number of genes by assembly length for publicly available Lepidoptera assemblies containing W and Z chromosomes and the HypMis_v2 and HypBol_v1. Pre- and Post-filtering refers to the assemblies after genes related to transposable elements have been filtered out. The complete dataset can be found in the Supplementary Information.


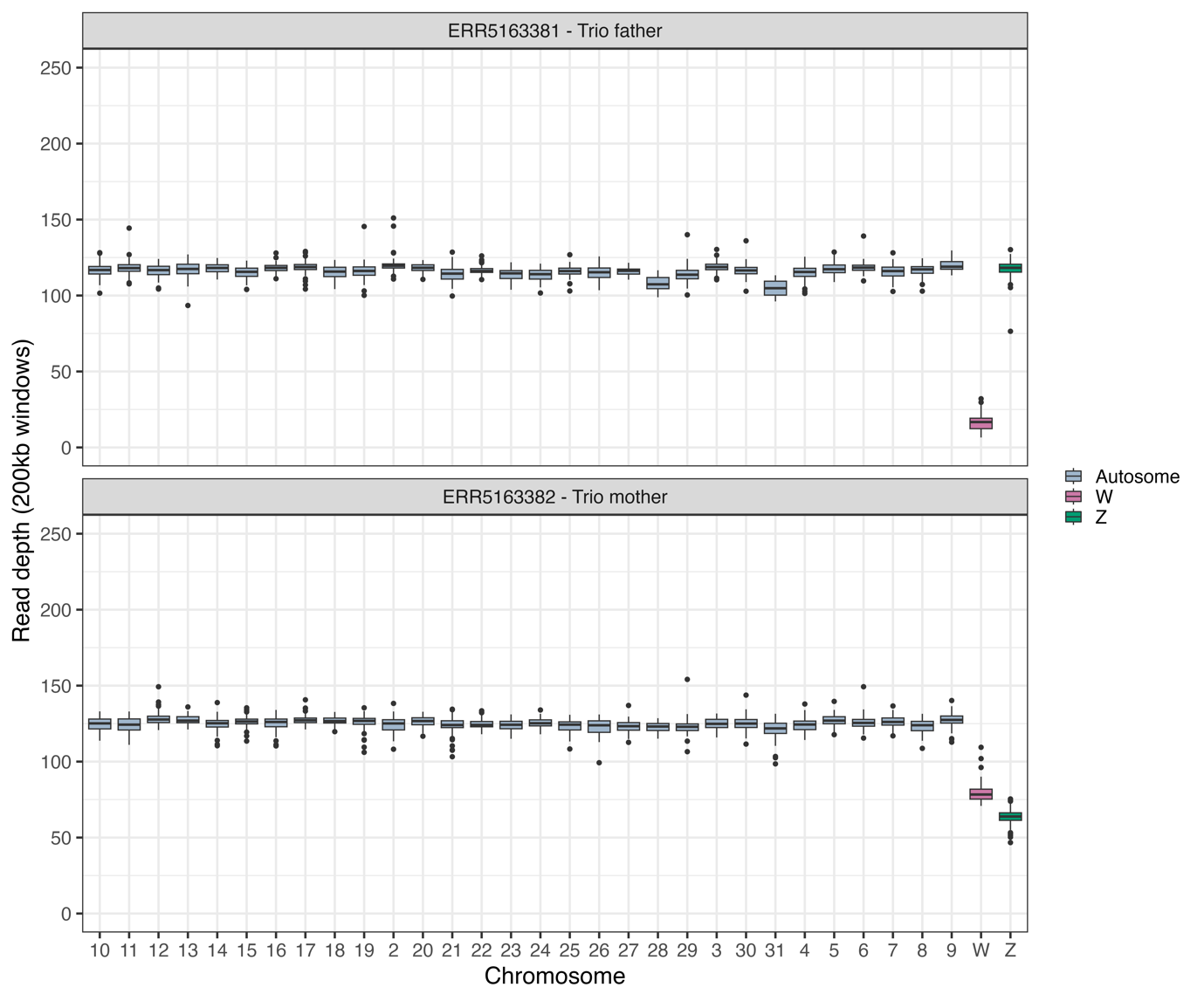


**Supplementary Figure 4.** Average read depth in 200kb windows for the mother and father of the trio reared for genome sequencing of *H. misippus.* Average read depth for the Z chromosome of the father is the same as for autosomes, while it is minimal for the W and probably only due to the high repeat content. Average read depth for the Z chromosome of the mother is about half of that of the autosomes, and a similar situation is seen for the W, albeit slightly higher.

**
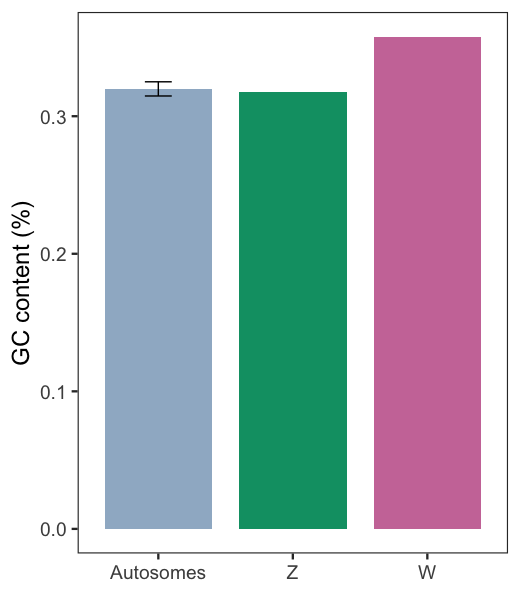
**

**Supplementary Figure 5.** GC content of the W chromosome of *H. misippus* is higher than Autosomes and Z, as seen in other Lepidoptera (Lewis et al. 2021; Lohse, Mackintosh, et al. 2021; Berner et al. 2023; Wan et al. 2019).


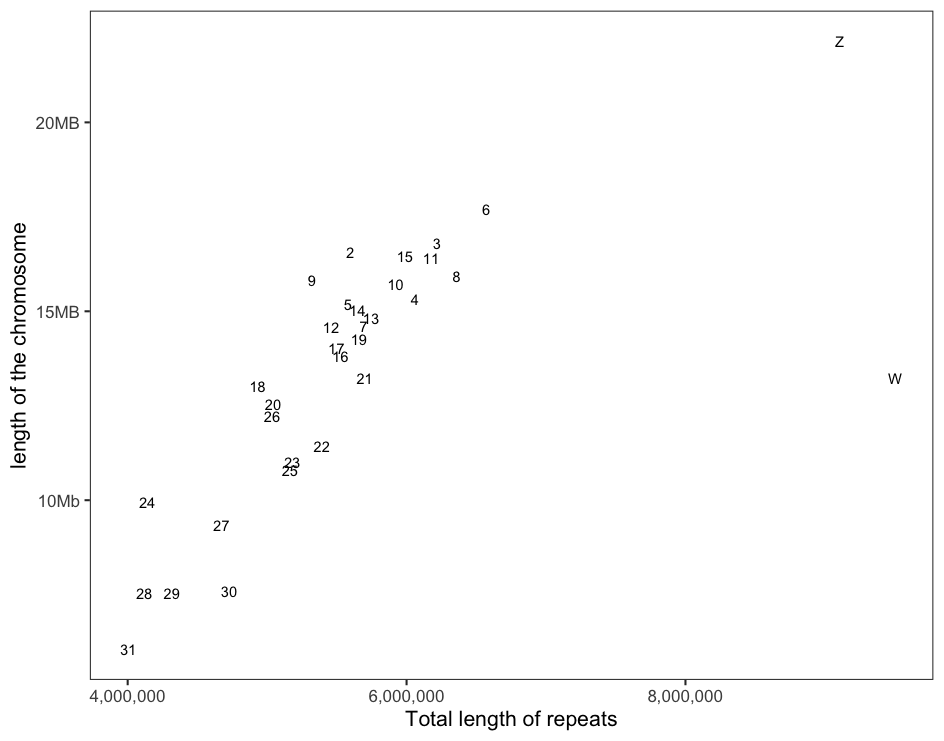


**Supplementary Figure 6.** Repeat content of the *HypMisi_v2* by chromosome. Larger chromosomes tend to have more repeats. The W chromosome deviates from the correlation and shows a higher repeat content for its length (see also Supplementary Figure 4).


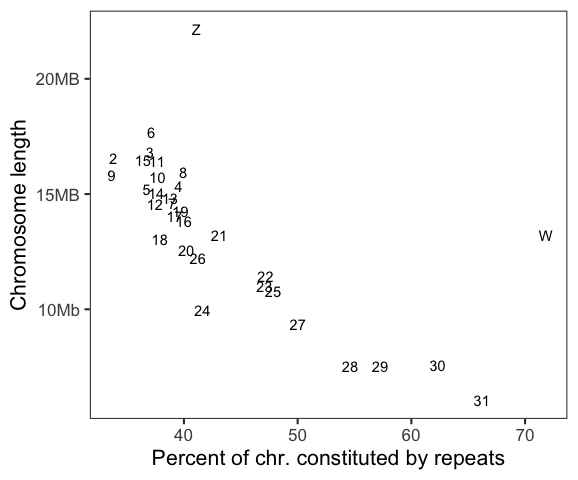


**Supplementary Figure 7.** Percentage of each chromosome of the *HypMisi_v2* assembly constituted by repeats. The W chromosome has the highest percentage of repeats.

**
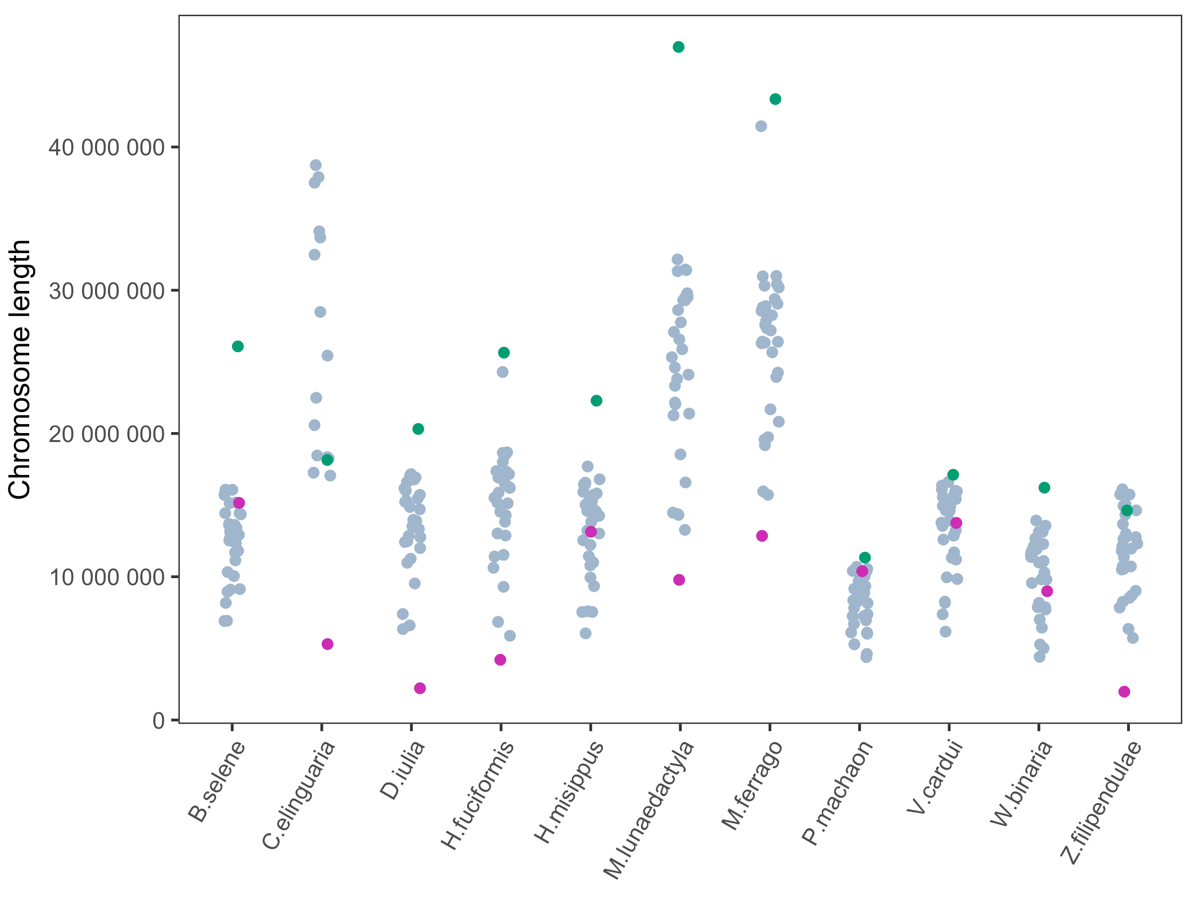
Supplementary Figure 8.** Chromosomes sizes of the 11 Lepidoptera species used for the comparisons. Autosomes are shown in grey, W chromosomes in pink and Z chromosomes in green.


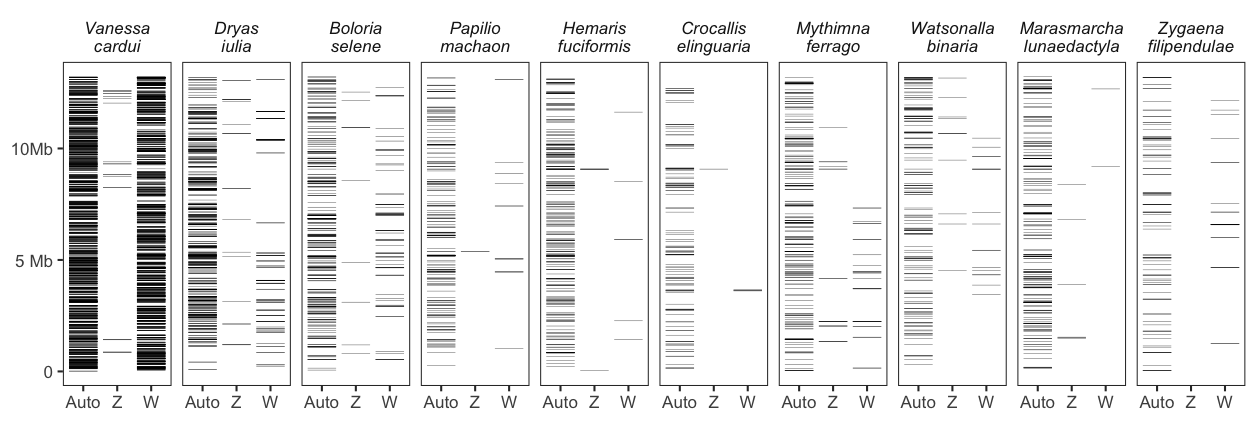


**Supplementary Figure 9.** The positions of synteny blocks between the *H. misippus* W chromosome and the chromosomes of other species do not reveal a conserved section of the W chromosome across species. Left-most column shows all synteny blocks with autosomes plotted.

**
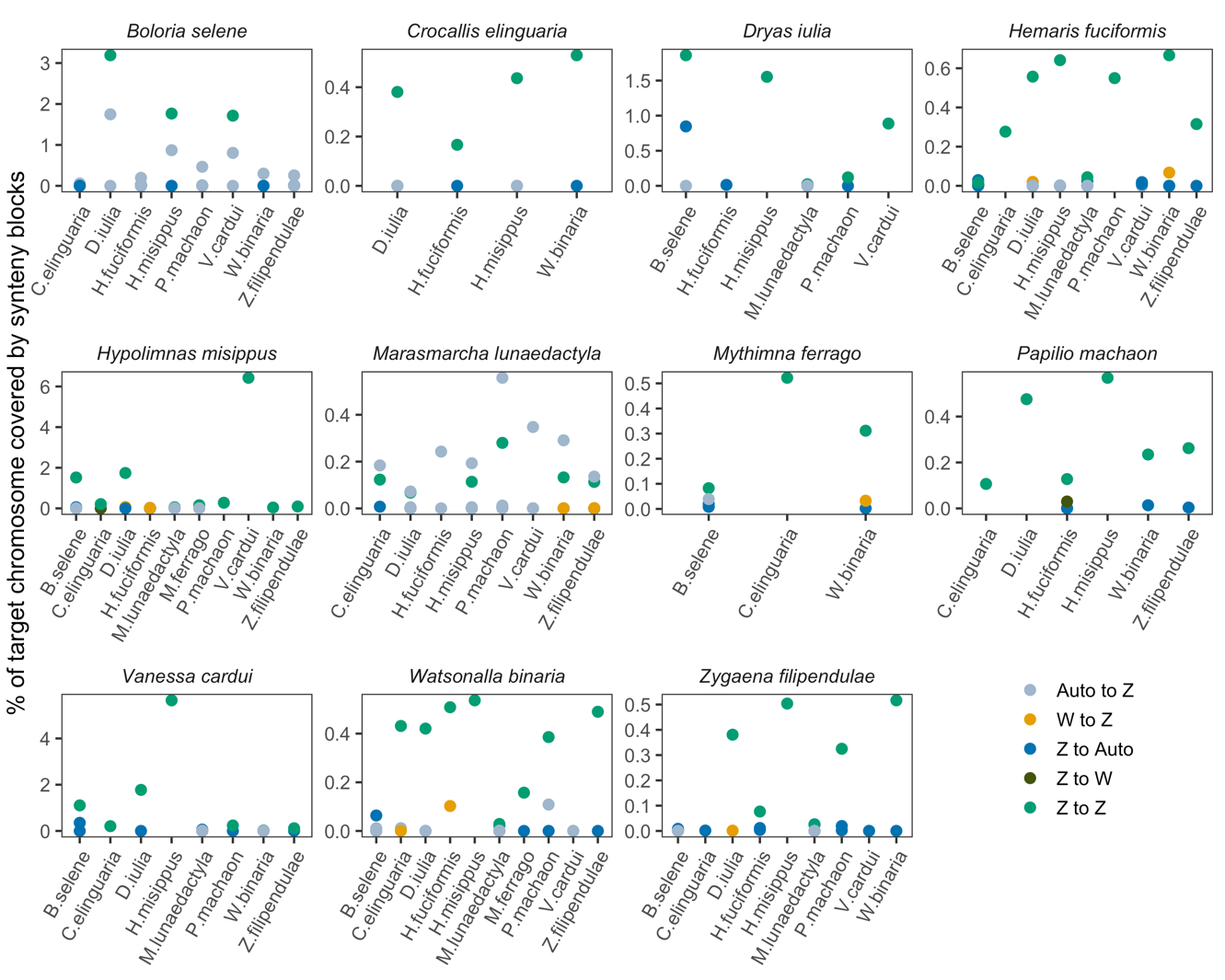
Supplementary Figure 10.** Synteny analysis using Satsuma2 between 11 ditrysian species reveals deep conservation of the Z chromosome across species. Each plot shows the result for a query species, that is the genome that has been used as query in the Satsuma analysis. X-axis shows the target species. The y-axis shows the percentage of target chromosome that is covered by query synteny blocks. Auto refers to any autosomes. Colours show the different possible results of which type of target chromosome maps to which type of query. For example “W to Z” refers to hits of the query W chromosome mapping to the Z target chromosome. Here, to evaluate the evolutionary history of the Z chromosome, only results involving the Z chromosome (as target or query) are shown.

**
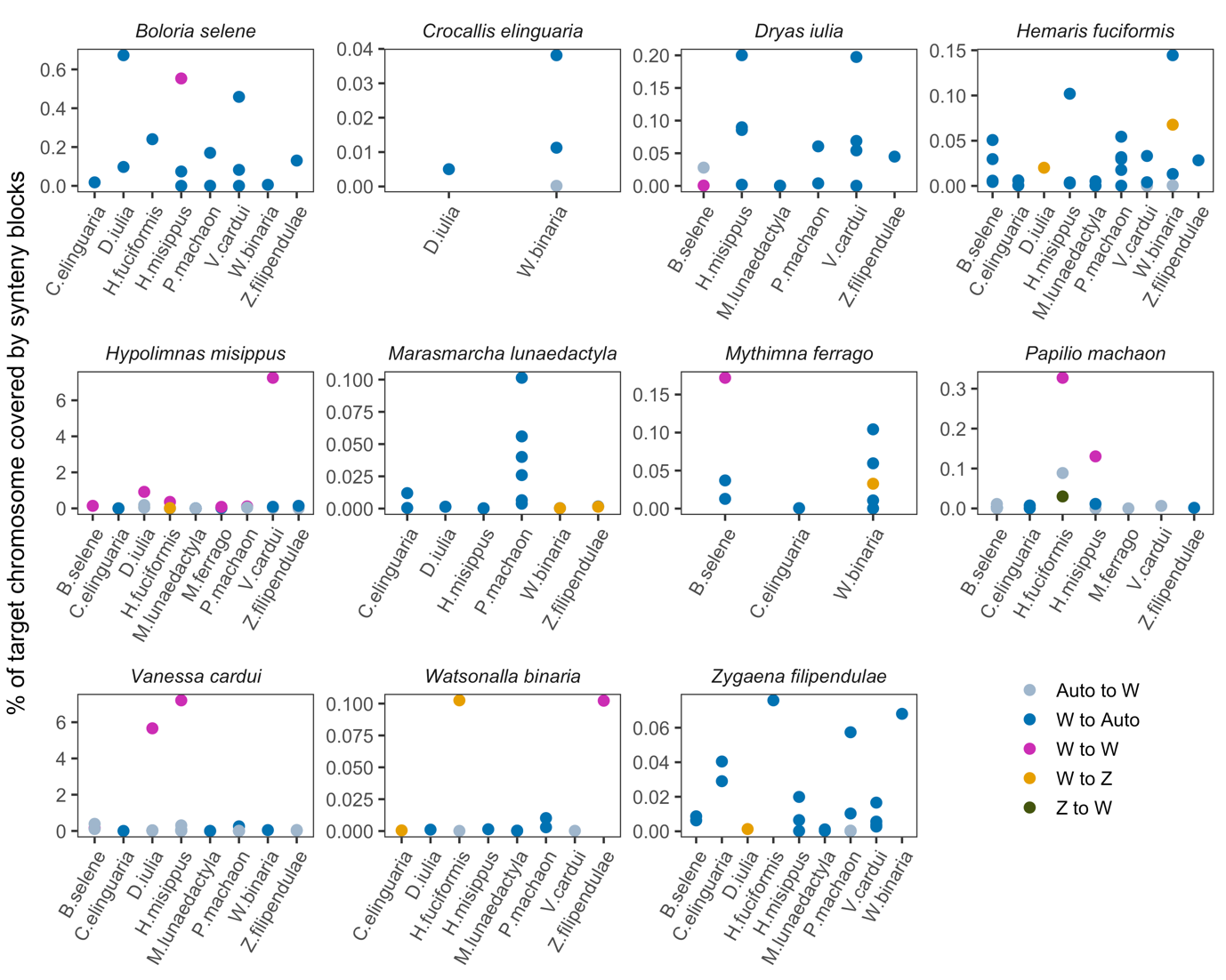
**

**Supplementary Figure 11.** Synteny analysis using Satsuma2 between 11 ditrysian species reveals some conservation of the W chromosome across species. Each plot shows the result for a query species, that is the genome that has been used as query in the Satsuma analysis. X-axis shows the target species. The y-axis shows the percentage of target chromosome that is covered by query synteny blocks. Auto refers to any autosomes. Colours show the different possible results of which type of target chromosome maps to which type of query. For example “W to Z” refers to hits of the query W chromosome mapping to the Z target chromosome. Here, to evaluate the evolutionary history of the W chromosome, only results involving the W chromosome (as target or query) are shown.


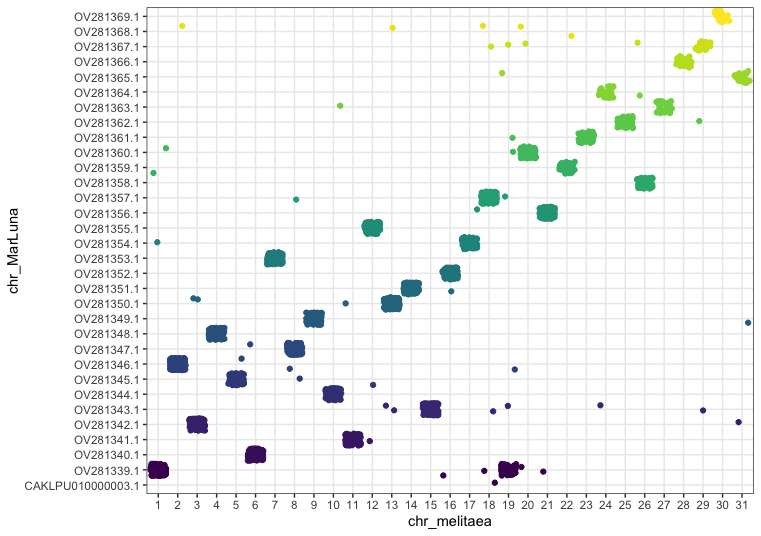


**Supplementary Figure 12.** Homology of *M. lunaedactyla’s* chromosomes with *Melitaea cinxia* reveals a neo-Z chromosome. Chromosome OV281339.1 of *M. lunaedactyla* shares BUSCOs with chromosome 1 (Z) and chromosome 19 of *M. cinxia*, suggesting a fusion of these two.


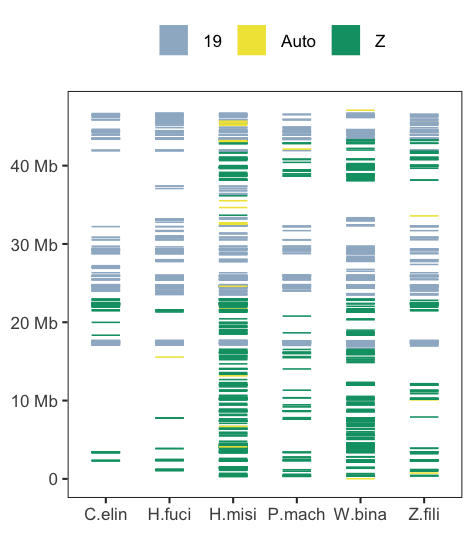


**Supplementary Figure 13.** Synteny analysis using Satsuma2 of the neo-Z chromosome of *M. lunaedactyla* compared to other ditrysian assemblies reveals the patterns of homology with the ancestral Z and chromosome 19.
